# Por Secretion System-Dependent Secretion and Glycosylation of *Porphyromonas gingivalis* Hemin-Binding Protein 35

**DOI:** 10.1371/journal.pone.0021372

**Published:** 2011-06-22

**Authors:** Mikio Shoji, Keiko Sato, Hideharu Yukitake, Yoshio Kondo, Yuka Narita, Tomoko Kadowaki, Mariko Naito, Koji Nakayama

**Affiliations:** 1 Division of Microbiology and Oral Infection, Department of Molecular Microbiology and Immunology, Nagasaki University Graduate School of Biomedical Sciences, Nagasaki, Japan; 2 Department of Pediatric Dentistry, Nagasaki University Graduate School of Biomedical Sciences, Nagasaki, Japan; 3 Global COE Program at Nagasaki University, Nagasaki, Japan; Monash University, Australia

## Abstract

The anaerobic Gram-negative bacterium *Porphyromonas gingivalis* is a major pathogen in severe forms of periodontal disease and refractory periapical perodontitis. We have recently found that *P. gingivalis* has a novel secretion system named the Por secretion system (PorSS), which is responsible for secretion of major extracellular proteinases, Arg-gingipains (Rgps) and Lys-gingipain. These proteinases contain conserved C-terminal domains (CTDs) in their C-termini. Hemin-binding protein 35 (HBP35), which is one of the outer membrane proteins of *P. gingivalis* and contributes to its haem utilization, also contains a CTD, suggesting that HBP35 is translocated to the cell surface via the PorSS. In this study, immunoblot analysis of *P. gingivalis* mutants deficient in the PorSS or in the biosynthesis of anionic polysaccharide-lipopolysaccharide (A-LPS) revealed that HBP35 is translocated to the cell surface via the PorSS and is glycosylated with A-LPS. From deletion analysis with a GFP-CTD[HBP35] green fluorescent protein fusion, the C-terminal 22 amino acid residues of CTD[HBP35] were found to be required for cell surface translocation and glycosylation. The GFP-CTD fusion study also revealed that the CTDs of CPG70, peptidylarginine deiminase, P27 and RgpB play roles in PorSS-dependent translocation and glycosylation. However, CTD-region peptides were not found in samples of glycosylated HBP35 protein by peptide map fingerprinting analysis, and antibodies against CTD-regions peptides did not react with glycosylated HBP35 protein. These results suggest both that the CTD region functions as a recognition signal for the PorSS and that glycosylation of CTD proteins occurs after removal of the CTD region. Rabbits were used for making antisera against bacterial proteins in this study.

## Introduction

*Porphyromonas gingivalis* is a black-pigmented, Gram-negative, asaccharolytic anaerobic bacterium. It is an etiologically important pathogen associated with adult periodontal disease [1], and it is thought to be associated with systemic illnesses including cardiovascular disease and rheumatoid arthritis [2], [3].

Considerable attention has been given to both characterizing the secreted and surface-associated proteins of *P. gingivalis* and determining their contributions to virulence. Among these, Arg-gingipains (Rgps) encoded by the *rgpA* and *rgpB* genes, Lys-gingipain (Kgp) encoded by the *kgp* gene, and hemagglutinins (Hag) encoded by the *hag* gene family [4], [5] are thought to be major virulence factors of *P. gingivalis*. The primary gene products of *rgpA*, *rgpB*, *kgp* and *hagA* contain a conserved C-terminal domain (CTD) consisting of approximately 80 amino acids that has been suggested to play a role in secretion and cell surface attachment [6]–[8]. The cell surface attachment of proteins such as RgpB appears to be linked to their glycosylation [7]–[9]. We have recently shown that the *hbp35* gene, which encodes a hemin-binding protein (HBP35) with one thioredoxin motif and a CTD, is transcribed as a monocistronic 1.1-kb mRNA, but it is subsequently translated into three discrete cytoplasmic proteins with molecular masses of 40, 29 and 27 kDa, and a diffuse cell surface protein with a molecular mass of 50–90 kDa [10]. The diffuse HBP35 protein reacts with the monoclonal antibody 1B5 (mAb 1B5), which recognizes a glycan epitope of anionic polysaccharides [11], [12]. These results suggested that the *P. gingivalis* HBP35 protein, like RgpB, is glycosylated on the cell surface.

The antibody mAb 1B5 recognizes a Manα1-2Manα1-phosphate side chain in anionic polysaccharides but not lipopolysaccharides (LPS; O antigen attached to lipid A core) or capsular polysaccharides [12], [13]. Because anionic polysaccharide was found to be linked to a lipid A core, it was recently renamed A-LPS (normal LPS is now called O-LPS) [14]. Our previous study showed that the *porR* gene, encoding a putative aminotransferase, plays a role in colony pigmentation on blood agar plates and that mAb 1B5 does not recognize any products in the *porR* mutant, suggesting that *porR* is involved in the biosynthesis of A-LPS [15]. Thereafter, mutant studies using *vimA*, *vimE*, *vimF* [16], [17], *wbpB* [18], *rfa* encoding a heptosyl transferase [19], *waaL* encoding an O-antigen ligase, *wzy* encoding an O-antigen polymerase [20] and *gtfB* [21] have shown that these genes are also involved in A-LPS biosynthesis. However, the mechanisms of A-LPS biosynthesis and of HBP35 protein binding to A-LPS remain to be determined.

We found a gene (named *porT*) that is responsible for the translocation of gingipains and HagA to the cell surface [22]. Since then, *sov* [23] and *pg27* [24] have been reported to contribute to gingipain secretion. We recently identified 11 genes (including *porT*, *sov* and *pg27*) that are involved in the secretion of gingipains and HagA and are designated the Por secretion system (PorSS) [25].

In this study, we characterized the secretion and glycosylation mechanism of HBP35 in *P. gingivlais* and found that HBP35 is transported by the PorSS and is glycosylated with A-LPS on the cell surface.

## Results

### Translational start site of the diffuse HBP35 protein

**Figure 1:**
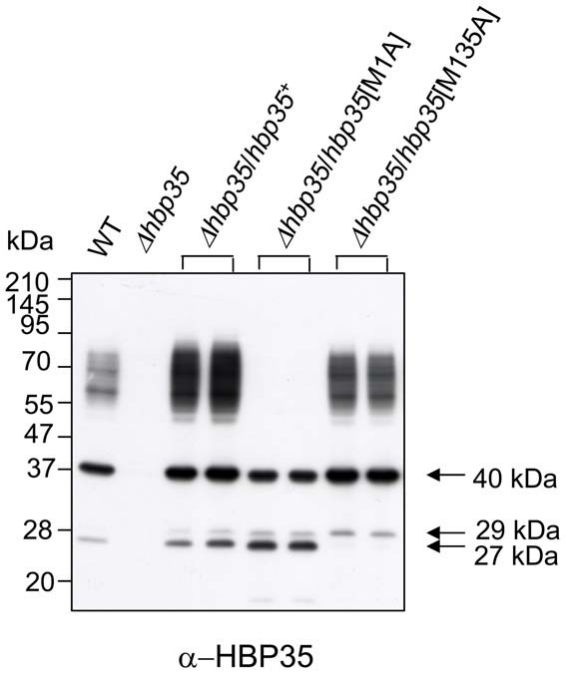
Effect of amino acid substitution of methionine residues on formation of the diffuse HBP35 protein. Cell lysates of *P. gingivalis* strains with amino acid substitutions of HBP35 Met^1^ or Met^135^ to Ala were subjected to SDS-PAGE and immunoblot analysis with anti-HBP35 antibodies.

A previous study showed that the *hbp35* gene generates various proteins with molecular masses of 50–90 (diffuse), 40, 29 and 27 kDa and that the 29- and 27-kDa proteins are translated from M^115^ and M^135^, respectively [10]. First, we determined the translational start site of the diffuse HBP35 proteins (UniProt accession number: Q8G962) (Figure 1). The diffuse HBP35 proteins completely disappeared in an M^1^A-substituted *hbp35* mutant strain, whereas the HBP35 proteins with molecular masses of 40, 29 and 27 kDa could still be detected in this mutant. These results suggested that the diffuse HBP35 protein is translated from M^1^. As the HBP35 protein translated from M^1^ contains a typical signal sequence (21 amino acids) at its N terminus, the diffuse protein appears to initially possess the signal peptide region, which is necessary for translocation across the cytoplasmic membrane. Interestingly, the 40-kDa HBP35 protein was detected in the mutant, suggesting that the 40-kDa protein is translated from an alternative initiation codon.

### PorSS-dependent translocation of the diffuse HBP35 protein

We have reported that RgpA, RgpB, Kgp and HagA proproteins are secreted to the cell surface by a newly found secretion system, PorSS [25]. These proteins are members of a class of proteins that contain a consensus C-terminal domain (CTD) and are called CTD proteins [7]. We recently showed that the *hbp35* gene encodes a CTD at its 3′-terminal region, and that the C-terminal 5 amino acids are required for formation of the diffuse HBP35 protein, suggesting that HBP35 is secreted via the PorSS [10].

**Figure 2:**
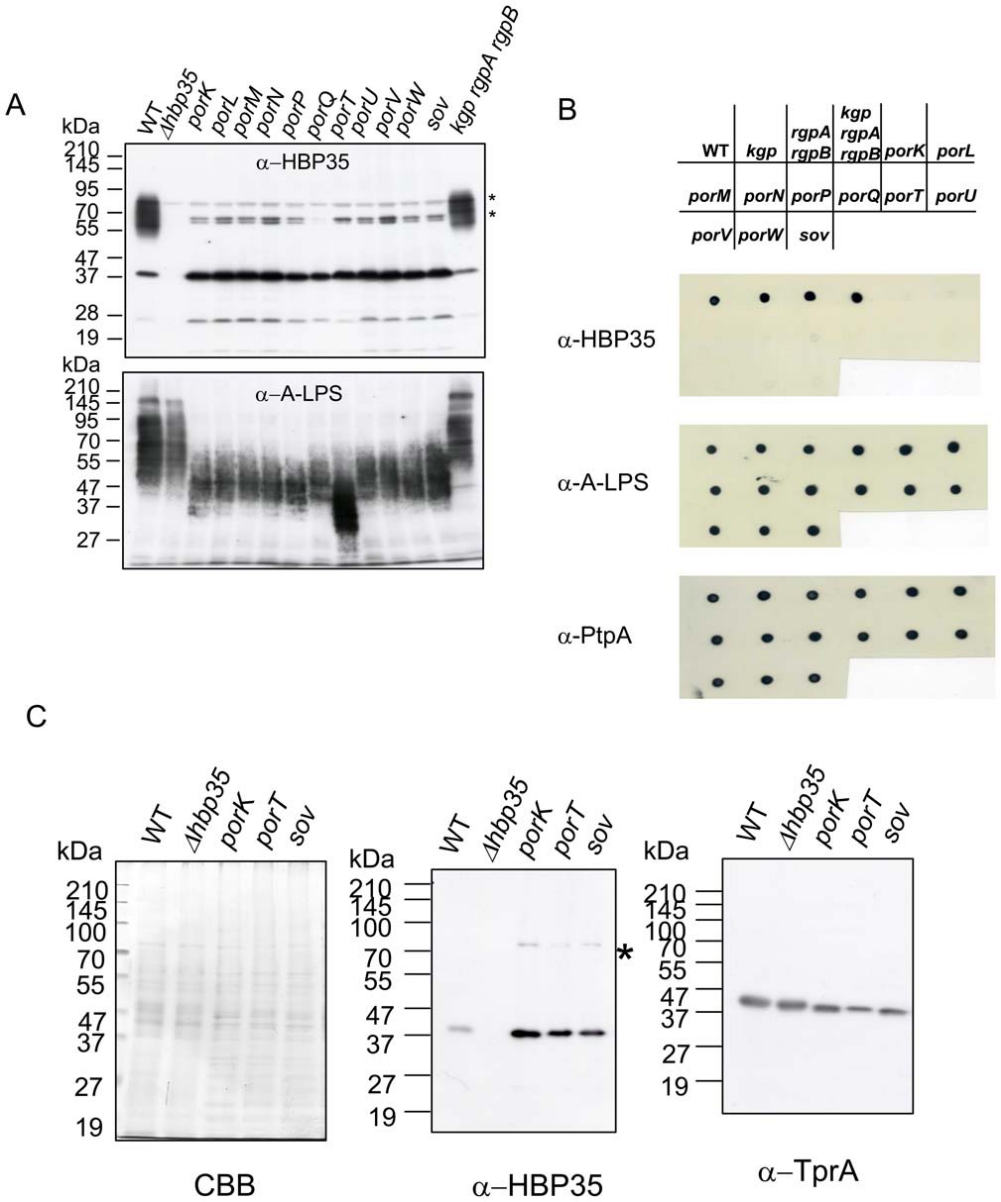
Por secretion system (PorSS)-dependent cell surface localization of HBP35. (A) Immunoblot analysis of *P. gingivalis* PorSS-deficient mutants with anti-HBP35 (upper) and anti-A-LPS (lower). (B) Dot blot analysis of *P. gingivalis* PorSS-deficient mutants with anti-HBP35 (upper), anti-A-LPS (middle) and anti-PtpA (lower). (C) Immunoblot analysis of the periplasmic fractions of PorSS-deficient mutants. Periplasmic fractions were subjected to SDS-PAGE and the resulting gel was stained with Coomassie Brilliant Blue (CBB) (left), or was immunoblotted with anti-HBP35 (middle) and anti-TprA (right). Asterisks indicate nonspecific cross-reactive protein bands.

To determine whether formation of the diffuse HBP35 protein depends on the PorSS, immunoblot analysis of 11 PorSS-deficient mutants (*porK*, *porL*, *porM*, *porN*, *porP*, *porQ*, *porT*, *porU*, *porV* (*pg27*, *lptO*) [24], [26], *porW* and *sov*) with anti-HBP35 antibodies was performed. All of the PorSS-deficient mutants examined had HBP35 with molecular masses of 40 and 27 kDa but lacked the diffuse protein (50–90 kDa) (Figure 2A). Because the diffuse HBP35 protein reacts with an anti-A-LPS monoclonal antibody (mAb 1B5) [10], we examined the PorSS-deficient mutants for the presence of A-LPS. Immunoblot analysis with an anti-A-LPS antibody revealed that the PorSS-deficient mutants had A-LPS; however, the molecular mass of A-LPS was less in the PorSS-deficient mutants than in wild-type cells (Figure 2A). We then determined whether HBP35 is present on the surface of PorSS-deficient mutant cells (Figure 2B). Dot blot analysis revealed that the intact cells of the 11 PorSS-deficient mutants blotted on a nitrocellulose membrane showed very weak reactivity with anti-HBP35 antibodies compared to PorSS-proficient strains. In contrast, the 11 PorSS-deficient mutants showed the same reactivity with anti-A-LPS and anti-prolyl tripeptidyl peptidase A (PtpA) antibodies as PorSS-proficient strains. PtpA, a cell surface protein, is secreted PorSS-independently [23], [24].

We previously reported that RgpA, RgpB, Kgp and HagA proproteins accumulate in the periplasm of a *porT* mutant [22]. Therefore, we asked whether HBP35 protein would also accumulate in the periplasm of *porT*, *porK* and *sov* mutants. As shown in Figure 2C, more amounts of the 40-kDa HBP35 protein were detected in periplasmic fractions of these mutants than in those of wild-type cells, whereas levels of the periplasmic protein TprA [27] were nearly identical in wild-type and mutant periplasmic fractions.

**Figure 3:**
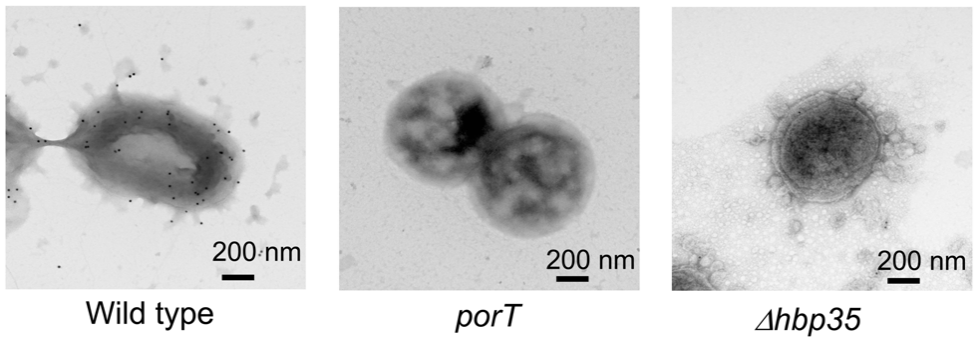
Immuno-electron microscopy with anti-HBP35-conjugated gold particles. Panel: left, 33277 (wild type); middle, KDP117 (*porT*); right, KDP166 (Δ*hbp35*).

Immuno-electron microscopy with anti-HBP35-conjugated gold particles revealed that a number of the gold particles were detected on and around the wild-type cells, but not on the *porT* mutant cells (Figure 3). These results strongly suggest that HBP35 is translocated onto the cell surface via PorSS.

### Anti-HBP35 immunoblot analysis of various mutants nonreactive to anti-A-LPS

**Figure 4:**
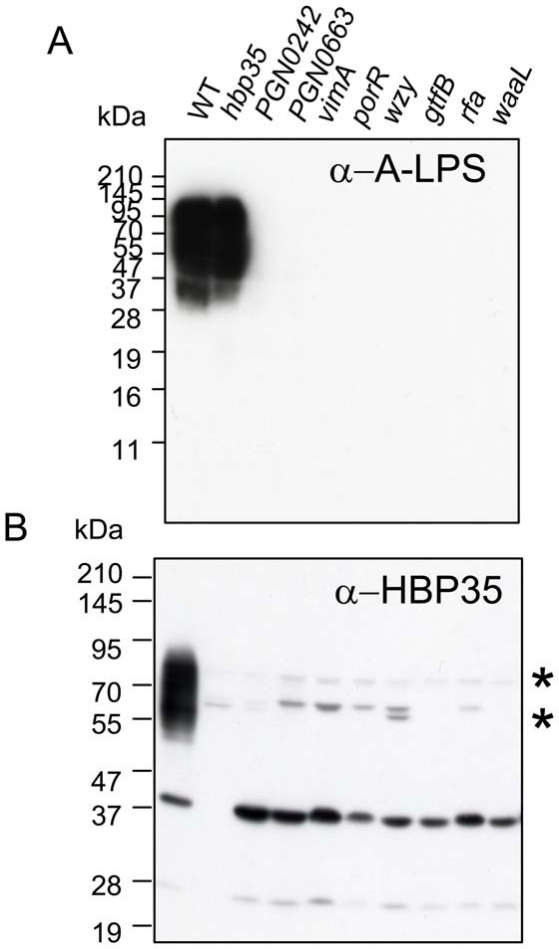
Immunoblot analysis of *P. gingivalis* A-LPS biosynthesis-deficient mutants with anti-A-LPS and anti-HBP35 antibodies. Cell lysates of *P. gingivalis* A-LPS biosynthesis-deficient mutants were subjected to SDS-PAGE and immunoblot analysis with anti-A-LPS and anti-HBP35 antibodies. Asterisks indicate nonspecific cross-reactive protein bands.

We previously suggested that PorR contributes to the biosynthesis of A-LPS, as cell lysates of the *porR* mutant show no reactivity with an anti-A-LPS antibody [15]. We have recently shown that partially purified diffuse HBP35 protein reacts with an anti-A-LPS antibody [10]. In addition to the *porR* mutant, *vimA*, *vimE*, *vimF*, *wbpB*, *waaL*, *wzy*, *rfa* and *gtfB* mutants have also been reported as nonreactive with an anti-A-LPS antibody [16]–[21]. We found in this study that PGN_0242 (encoding a putative mannosyl transferase), PGN_0663 (encoding a hypothetical protein), PGN_1056 (*vimA*), PGN_1236 (*porR*), PGN_1242 (*wzy*), PGN_1251 (*gtfB*), PGN_1255 (*rfa*) and PGN_1302 (*waaL*) mutants all show nonreactivity to an anti-A-LPS antibody (Figure 4A). To determine whether the diffuse HBP35 protein is present in the anti-A-LPS-nonreactive mutants, we carried out immunoblot analysis of the anti-A-LPS-nonreactive mutants with anti-HBP35 antibodies. All the mutants lacked the diffuse HBP35 protein (Figure 4B), suggesting that the diffuse HBP35 protein is bound to A-LPS.

### Construction and expression of the GFP-CTD fusion proteins in *P. gingivalis*

**Figure 5:**
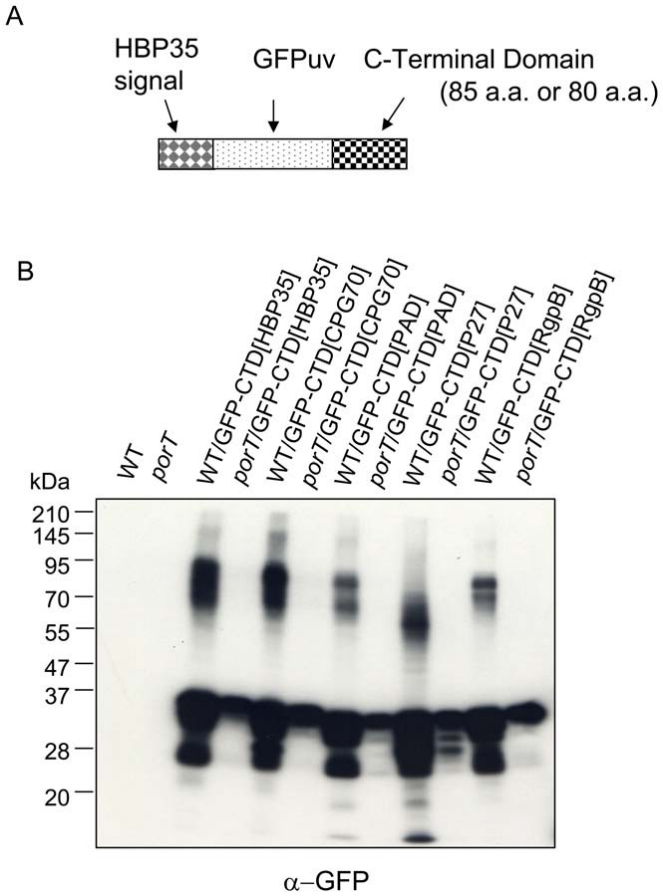
PorT-dependent glycosylation of GFP-CTD fusion proteins. (A) Diagram of a GFP-CTD fusion protein expressed in *P. gingivalis*. The fusion protein consists of a signal sequence from HBP35, a GFP domain and a CTD. An 85 amino acid C-terminal region containing the CTD was used for HBP35, CPG70, peptidylarginine deiminase (PAD) and P27, and one with 80 amino acids was used for RgpB. (B) Anti-GFP immunoblot analysis of *P. gingivalis* strains 33277 (WT) and KDP117 (*porT*) expressing GFP-CTD fusion proteins.

Several reports have suggested that the CTD of RgpB plays roles in secretion and cell-surface attachment [7]–[9]. To determine whether the CTDs of CTD proteins (including HBP35) play roles in secretion and cell-surface attachment, fusion proteins consisting of the HBP35 signal sequence region, green fluorescent protein (GFP) and CTDs of various CTD proteins were constructed and expressed in *P. gingivalis* cells (Figure 5A). *P. gingivalis* cells expressing fusion genes encoding GFP proteins fused to the C-terminal 85 amino acid residues of HBP35 (PGN_0659), CPG70 (PGN_0335), peptidylarginine deiminase (PAD; PGN_0898), P27 (PGN_1770) and GFP protein fused to the C-terminal 80 amino acid residues of RgpB (PGN_1466) produced anti-GFP-reactive diffuse protein bands in a wild-type background but not in a *porT* mutant (Figure 5B).

### Identification of a minimal CTD region required for formation of diffuse GFP-CTD[HBP35] fusion proteins

**Figure 6:**
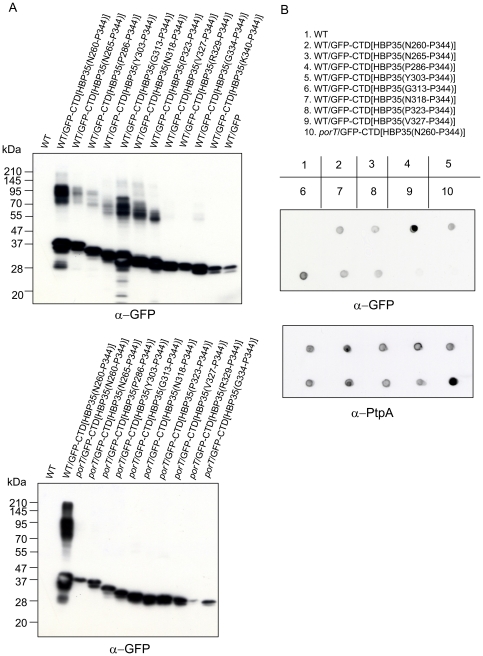
Identification of a minimal region of the HBP35 CTD required for glycosylation and cell surface localization. (A) Immunoblot analysis of GFP-CTD[HBP35] fusion proteins with nested deletions produced in *P. gingivalis* 33277 (WT) and KDP117 (*porT*). Cell lysates of wild-type and *porT P. gingivalis* strains expressing various GFP-CTD[HBP35] fusion proteins were subjected to SDS-PAGE and immunoblot analysis with anti-GFP antibodies. (B) Dot blot analysis of *P. gingivalis* expressing GFP-CTD[HBP35] fusion proteins with nested deletions. Intact *P. gingivalis* cells were blotted onto a nitrocellulose membrane and detected with anti-GFP and anti-PtpA antibodies.

**Figure 7:**
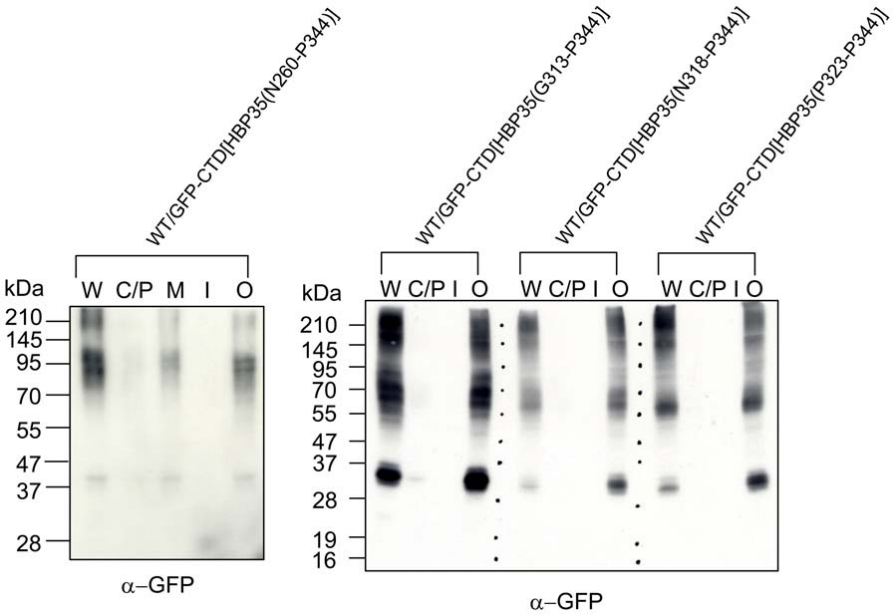
Cell fractionation analysis of *P. gingivalis* expressing GFP-CTD[HBP35] fusion proteins. *P. gingivalis* wild-type cells expressing GFP-CTD[HBP35(N^260^-P^344^)], GFP-CTD[HBP35(G^313^-P^344^)] or GFP-CTD[HBP35(P^323^-P^344^)] were fractionated into whole cell lysate (W), a cytoplasm/periplasmic fraction (C/P), a total membrane fraction (M), an inner membrane fraction (I) and an outer membrane fraction (O).

Next, we determined the minimal region of the HBP35 CTD required for the formation of diffuse GFP fusion protein bands. The C-terminal 22 amino acid residues of CTD[HBP35] were required for the formation of diffuse GFP fusion protein bands in a *porT*-dependent manner (Figure 6A). Approximately 20 amino acid residues of the CTD C-terminus are highly conserved [7], [8]. Dot blot analysis with anti-GFP antibodies revealed that the C-terminal 22 amino acid residues of the HBP35 CTD were required for cell surface localization of the GFP fusion protein in a *porT*-dependent manner (Figure 6B). Furthermore, cell fractionation analysis revealed that the diffuse GFP fusion proteins were localized in the outer membrane fraction (Figure 7). These results suggest that the highly conserved C-terminal region (approximately 20 amino acid residues) of the CTD plays an important role in translocation to the cell surface via the PorSS.

**Figure 8:**
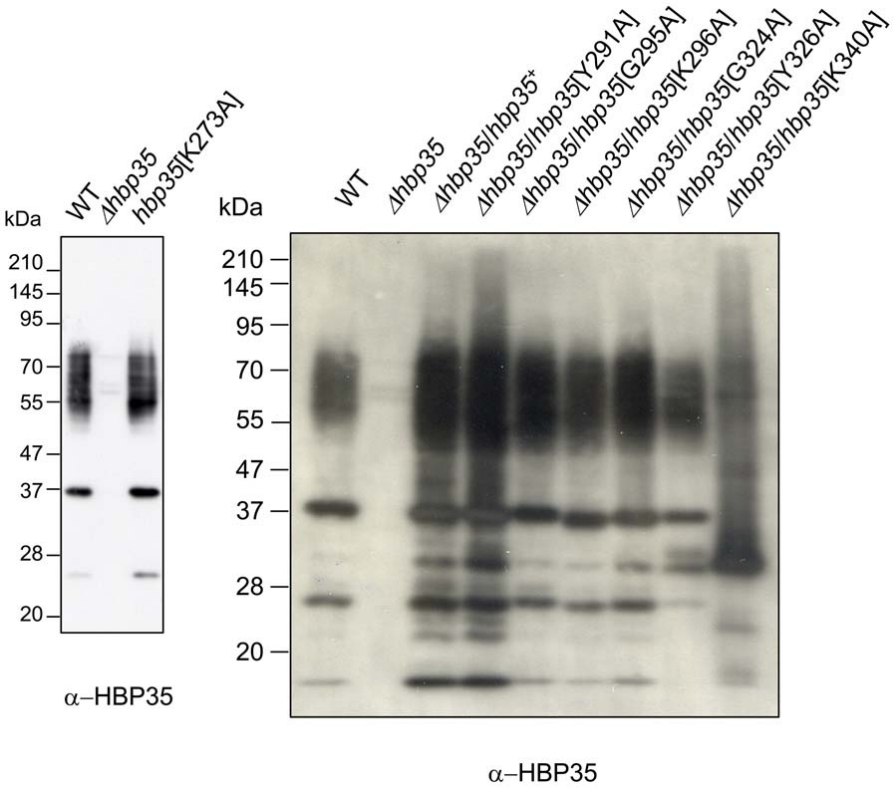
Substitution of various amino acid residues in the CTD of HBP35. Cell lysates of *P. gingivalis* expressing HBP35 proteins with substitutions of various amino acid residues in the CTD region were subjected to SDS-PAGE and immunoblot analysis with anti-HBP35 antibody.

**Figure 9:**
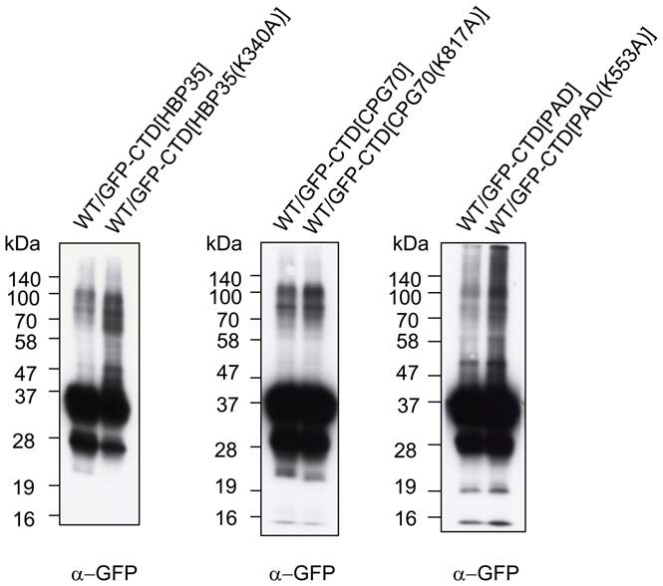
Immunoblot analysis of *P. gingivalis* expressing GFP-CTD fusion proteins with or without Lys substitution. Cell lysates of *P. gingivalis* expressing GFP-CTD[HBP35], GFP-CTD[CGP70] or GFP-CTD[PAD] fusion proteins with or without substitution of Lys^340^, Lys^817^ or Lys^553^ to Ala, respectively, were subjected to SDS-PAGE and immunoblot analysis with anti-GFP antibody.

Next, we attempted to identify amino acid residues in the CTD of HBP35 that are required for formation of the diffuse HBP35 protein. We constructed *P. gingivalis* strains expressing HBP35 protein with the following substitutions: K^273^A, Y^291^A, G^295^A, K^296^A, G^324^A, Y^326^A and K^340^A. The K^273^A, Y^291^A, G^295^A, K^296^A, G^324^A and Y^326^A substitutions showed no effect on the diffuse protein bands, whereas the K^340^A substitution showed a different pattern (Figure 8). As substitution of the corresponding K of RgpB (K^503^) had no effect on the diffuse RgpB protein in a previous study [8], we constructed mutant strains with substitutions of the corresponding Lys residues of GFP-CTD[HBP35], GFP-CTD[CPG70] and GFP-CTD[PAD], and performed immunoblot analysis of the mutants with anti-GFP antibodies (Figure 9). *P. gingivalis* strains expressing the GFP-CTD fusion proteins with the Lys substitutions produced diffuse bands just as the parent strains did, suggesting that this Lys residue is not essential for the formation of the diffuse CTD proteins.

### Peptide map fingerprinting (PMF) and immunoblot analyses of the diffuse HBP35 protein

**Figure 10:**
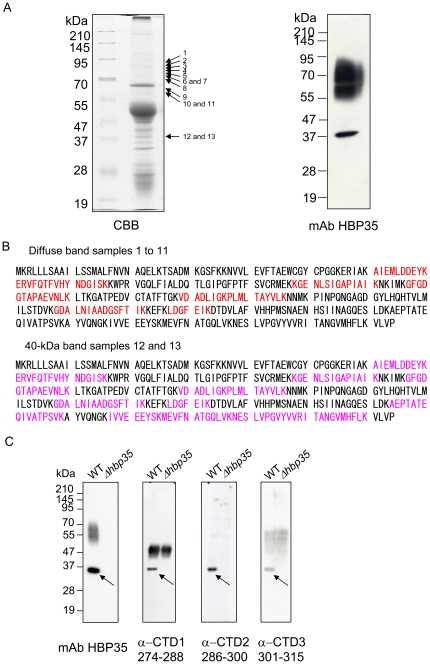
Peptide map fingerprinting (PMF) and immunoblot analyses of the diffuse HBP35 protein. (A) CBB staining and anti-HBP35 immunoblotting of anti-HBP35 immunoprecipitates from *P. gingivalis* cell lysates. Immunoprecipitates from the cell lysates of the *rgpA rgpB kgp* mutant were separated by SDS-PAGE, and the resulting gel was stained with CBB (left panel) or probed with a mAb against HBP35, mAb-Pg ompA2 (right panel). Arrows indicate the positions of samples 1 to 11 collected from the diffuse HBP35 protein bands and those of samples 12 and 13 collected from the 40-kDa HBP35 protein band. (B) PMF analysis. Peptide fragments (red in the upper sequence) were identified in the 11 samples of the diffuse HBP35 protein, and peptide fragments (pink in the lower sequence) identified in the 2 samples of the 40-kDa HBP35 protein. (C) Immunoblot analysis of *P. gingivalis* with antibodies against peptides in the CTD region of HBP35. Cell lysates of *P. gingivalis* 33277 (WT) and KDP166 (Δ*hbp35*) were subjected to SDS-PAGE and immunoblot analysis with an mAb against HBP35, anti-CTD1 [A^274^-V^288^], anti-CTD2 [P^286^-E^300^] and anti-CTD3 [E^301^-L^315^]. Arrows indicate the 40-kDa HBP35 protein.

Veith et al. [6], [28] identified 18 CTD-containing proteins in the outer membrane fraction of *P. gingivalis* by PMF analysis. That report did not identify any peptide fragments from within the CTDs of the 18 CTD-containing proteins. We sought to identify peptide fragments of the diffuse HBP35 protein and the 40-kDa HBP35 protein. Cell lysate of the *rgpA rgpB kgp* mutant (KDP136) was immunoprecipitated with anti-HBP35 antibodies and separated by SDS-PAGE. The resulting gel was cut and subjected to in-gel digestion by trypsin followed by LC-MS/MS (Figure 10A). Peptide fragments of the diffuse HBP35 protein and the 40-kDa HBP35 protein, which were located at C-terminal most, were L^237^-K^243^ and I^330^-K^340^, respectively (Figure 10B, Table S1).

To determine whether the diffuse HBP35 protein contains the CTD region, we made mouse antibodies using peptides (A^274^-V^288^, P^286^-E^300^ and E^301^-L^315^) derived from the CTD region of HBP35. All of the peptide antibodies reacted with the 40-kDa HBP35 protein but not the diffuse HBP35 protein (Figure 10C). Immunoreactive diffuse protein bands were found in immunoblots of wild-type cells with anti-CTD1 and anti-CTD3, but the same protein bands were also detected in an *hbp35* deletion mutant, suggesting that they are non-specific.

## Discussion

We recently found that a new secretion apparatus, termed the Por secretion system (PorSS), exists in *P. gingivalis* [25]. As RgpA, RgpB, Kgp and HagA of *P. gingivalis*, which are secreted via the PorSS, have conserved CTDs, it seems likely that the CTD plays a critical role in PorSS-mediated secretion. The present study clearly shows that HBP35 is transported to the cell surface via the PorSS (Figure 2A and B). We previously found that unprocessed CTD proteins including RgpA, RgpB and Kgp accumulate in the periplasm of the PorSS-deficient mutant *porT* [22]. Similarly, the 40-kDa HBP35 protein was more abundant in the periplasm of the PorSS-deficient mutants *porT*, *porK* and *sov* than in that of wild-type cells (Figure 2C). As the 40-kDa HBP35 protein is not glycosylated [10] and A-LPS is present on the surface of PorSS-deficient mutant cells (Figure 2B), the glycosylation event appears to occur not in the periplasm but on the outer membrane.

As shown in Figure 5B, genetic fusion of *gfp* to the CTD-encoding DNA region of either *p27*, *cpg70*, *pad*, *rgpB* or *hbp35* caused diffuse GFP protein to appear in a PorT-dependent manner, suggesting that P27, CGP70 and PAD are also secreted via the PorSS. P27 is one of the major outer membrane proteins and appears as a diffuse protein in the molecular mass range of 43–64 kDa that reacts with an anti-A-LPS antibody [6]. CPG70 has a carboxyl peptidase activity and the *cpg70* mutant is less virulent [29]. PAD catalyzes the citrullination of arginine residues that are located at the C-termini of peptides possibly generated by Rgp digestion, and it is thought to be related to the progression of rheumatoid arthritis [30], suggesting that CTD proteins transported by PorSS play important roles in the virulence of *P. gingivalis*.

Seers et al. [7] suggested that the CTD contains 5 motifs designated A, B, C, D, and E. Nested deletion of the CTD region of HBP35 in the GFP-CTD[HBP35] fusion protein revealed that the C-terminal 22 amino acid residues containing the D and E motifs are required for transport of the GFP-CTD[HBP35] fusion protein to the cell surface and that this transport is dependent on PorT (Figure 6). The D and E motifs are well conserved not only in CTD proteins of *P. gingivalis*, *Tannerella forsythia* and *Prevotella intermedia* [8] but also in the C-terminal domains of some PKD1-homologous proteins in eukaryotes [31]. Mutations in PKD1 are assumed to be related to autosormal-dominant polycystic kidney disease. The C-terminal domain of the PKD1 protein is predicted to form an anti-parallel β-sheet structure, suggesting that the C-terminal region of HBP35 including the D and E motifs also forms an anti-parallel β-sheet structure and play a critical role in recognition by PorSS. Although A, B and C motifs of the CTD region are not necessary for secretion of the GFP-CTD fusion protein, the motifs may be important for secretion of *P. gingivalis* CTD proteins by preventing protein folding in the periplasm prior to secretion. In Figure 6, the observed molecular masses of non-modified GFP-CTD fusion proteins correlated with the calculated ones. On the other hand, the change in molecular masses of the diffuse GFP-CTD fusion proteins did not correlate with the change in size of the CTD regions in the fusions, suggesting that post-translational glycosylation varies among the GFP proteins fused with various lengths of the CTD domain.

*P. gingivalis* produces at least three polysaccharide molecules on its surface: O-LPS (O antigen attached to a lipid A core) [20], A-LPS (phosphorylated branched mannan repeat units attached to a lipid A core) [14] and capsular polysaccharides [13]. Curtis et al. [11] raised a monoclonal antibody (mAb 1B5) against the catalytic domain of RgpA; it cross-reacts with A-LPS and recognizes phosphorylated branched mannan in the anionic polysaccharide repeating unit [12]. Our previous study showed that the *porR* gene, encoding a putative aminotransferase, is involved in anchoring gingipains to the cell surface [15]. We found in that study that mAb 1B5 did not recognize any products of the *porR* mutant. Neither *vimA* [16], *rfa* [19], *waaL* [20], *wzy* [20] nor *gtfB* mutants [21] are recognized by mAb 1B5, suggesting that these mutants lack A-LPS. The diffuse HBP35 protein is also lacking in these mutants (Figure 4), which is consistent with the previous result that purified diffuse HBP35 protein reacts with mAb 1B5 [10].

**Figure 11:**
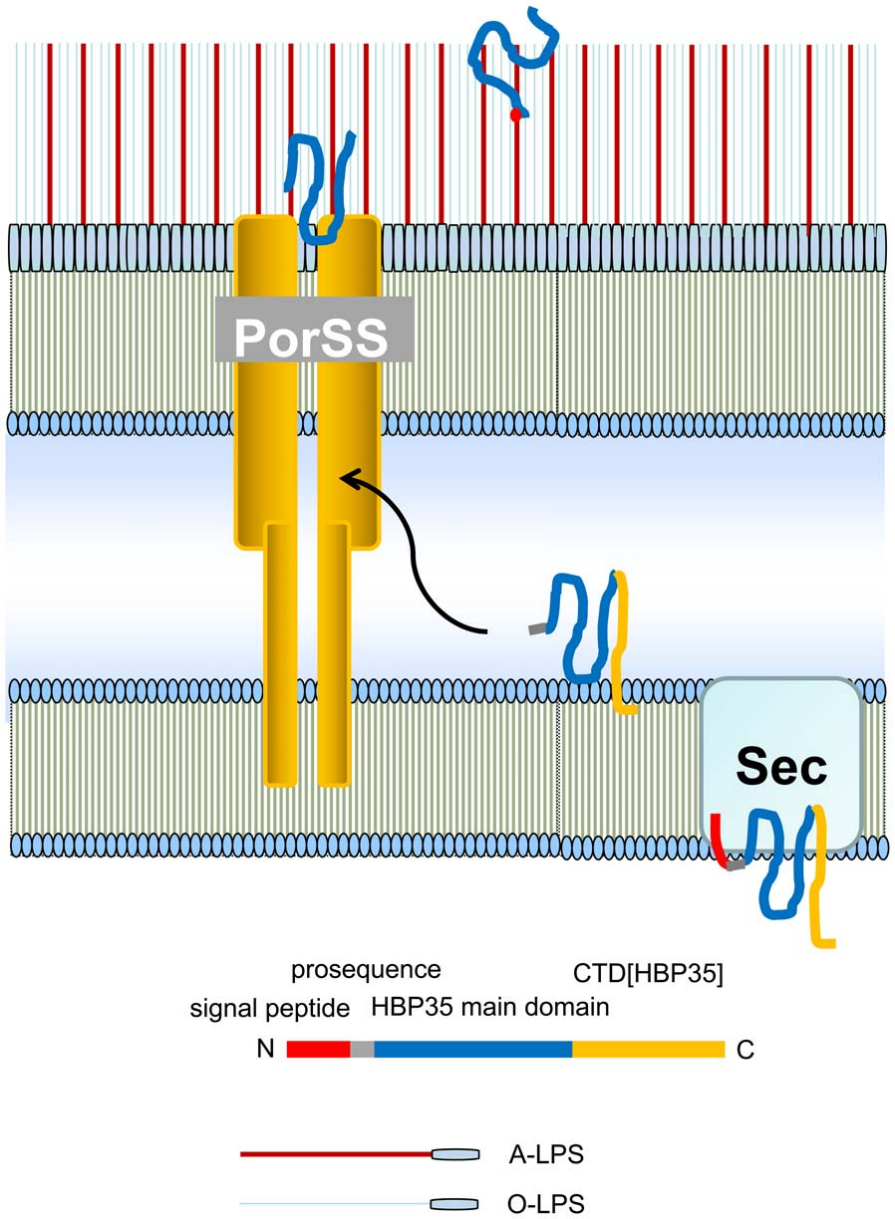
Model for the transport and glycosylation mechanism of the HBP35 protein in *P. gingivalis*.

A search of the *P. gingivalis* protein database revealed that CTDs consisting of approximately 80 amino acid residues are present in 34 proteins [6]. Eighteen of these 34 CTD proteins have been experimentally shown to be located in the outer membrane fraction of *P. gingivalis* [7]. In *T. forsythia*, 13 of 26 CTD-family proteins identified by in silico analysis were found to be outer membrane proteins by MALDI-TOF/TOF analysis [27]. Interestingly, no peptides derived from the CTD region were detected in any of the CTD proteins found in the outer membrane fraction by MS/MS analysis [6], [27]. In the present study, peptides derived from the CTD region of HBP35 were detected in the 40-kDa HBP35 protein but not in the diffuse HBP35 protein (Figure 10B). Moreover, antibodies against peptides within the CTD region of HBP35 recognize the 40-kDa HBP35 protein but not the diffuse HBP35 protein (Figure 10C). These results suggest that the CTD region is not present in the diffuse HBP35 protein; however, we cannot exclude the possibility that glycan modification in the CTD region hampers both immune reactivity with anti-CTD-peptide antibodies and the detection of peptide fragments from the CTD region by mass spectrometric analysis. If CTD proteins on the cell surface lack their CTD region, it is likely that the CTD region is removed during transport by PorSS or on the cell surface and that there, CTD proteins lacking their CTD region are glycosylated with A-LPS (Figure 11).

In eukaryotes, glycosylated proteins are common components of extracellular matrices and cellular surfaces. Their oligosaccharide moieties are implicated in a wide range of cell-cell and cell-matrix recognition events that are required for biological processes ranging from immune recognition to cancer development. Glycoproteins are less common in prokaryotes than in eukaryotes; however, several studies of glycoproteins have been reported in prokaryotes. *Bacteroides fragilis*, which is a gut bacterium and belongs to the *Bacteroidetes* phylum but lacks PorSS [25], has a number of glycosylated proteins on its surface that are formed by an O-linked glycosylation pathway [32]. In general, there are two families of conjugating enzymes in Gram-negative bacteria: the N-linked oligosaccharyltransferases, well studied in *Campylobacter jejuni* PglB, and the O-linked oligosaccharyltransferases, including *Neisseria meningitidis* PglL [33]. These enzymes work on the periplasmic side of the inner membrane. In particular, it has been shown that PglB functions not only as an N-linked oligosaccharyltransferase but also as an N-linked O-antigen transferase [34], which has a role similar to the O-antigen ligase (WaaL) responsible for attaching O-antigen to the lipid A-core. Rangarayan et al. [14] reported that *P. gingivalis* WaaL contributes to the binding of the two polysaccharide repeating units to the lipid A core. *P. gingivalis* WaaL also contributes to the biosynthesis of A-LPS and the formation of the diffuse HBP35 protein ([20]; this study, Figure 4). However, it is unknown whether the *P. gingivalis* WaaL protein plays a role in binding CTD proteins to A-LPS. Very recently, Slakeski et al. [9] have reported that the Ser/Thr/Tyr/Asn residues of the CTD region of RgpB are not the sites of modification and surface attachment, as revealed by site-directed mutagenesis. Treatment of the diffuse HBP35 protein with either an N-linked glycanase, PNGase F or some O-linked glycanases failed to alter the molecular masses of the diffuse protein (data not shown). Moreover, no genes encoding N-linked or O-linked oligosaccharyltransferase homologs can be found in the *P. gingivalis* genome, suggesting that the binding of CTD proteins to A-LPS is not catalyzed by N-linked or O-linked oligosaccharyltransferases.

OMP85 and Mfa1 of *P. gingivalis* have been reported to be glycosylated [35], [36]. These proteins, which are located in the outer membrane, do not have CTD regions and exhibit discrete protein bands (not diffuse bands) on a gel, suggesting that the glycosylation of these proteins may be different from that of CTD proteins, as CTD proteins such as RgpB, TapA, HBP35 and CPG70 all produce diffuse protein bands on a gel [8], [10], [26], [27].

In conclusion, the C-terminal 22 amino acid residues of CTD[HBP35] were found to be required for cell surface translocation and glycosylation from deletion analysis with the GFP-CTD[HBP35] protein fusion. The GFP-CTD fusion study also revealed that the CTDs of CPG70, peptidylarginine deiminase, P27 and RgpB play roles in PorSS-dependent translocation and glycosylation. Peptide map fingerprinting and immunoblot analyses suggest both that the CTD region functions as a recognition signal for the PorSS and that glycosylation of CTD proteins occurs after removal of the CTD region.

## Materials and Methods

### Bacterial strains and plasmids

The bacterial strains and plasmids used in this study are listed in Tables S2 [37]–[39] and S3 [40], respectively.

### Media and conditions for bacterial growth

*P. gingivalis* strains were grown under anaerobic conditions (80% N_2_, 10% CO_2_, 10% H_2_) in enriched brain-heart infusion (BHI) broth (Becton Dickinson) or on enriched Trypto-soya (TS) agar plates (Nissui) supplemented with hemin (5 µg/ml) and menadione (0.5 µg/ml). Luria-Bertani (LB) broth and LB agar plates were used to grow of *E. coli* strains. Antibiotics were used at the following concentrations: ampicillin (Ap; 100 µg/ml for *E. coli*, 10 µg/ml for *P. gingivalis*), erythromycin (Em; 10 µg/ml for *P. gingivalis*), gentamicin (Gm; 50 µg/ml for *E. coli*) and tetracycline (Tc; 0.7 µg/ml for *P. gingivalis*).

### Chemicals

The proteinase inhibitors Nα-p-tosyl-_L_-lysine chloromethyl ketone (TLCK) and iodoacetamide were purchased from Wako, and leupeptin was obtained from Peptide Institute.

### Construction of *P. gingivalis* strains

The oligonucleotides used in this study are listed in Table S4. General manipulation of DNA, restriction enzyme digestion, plasmid mapping and transformation of *E. coli* and *P. gingivalis* have been described in detail [10]. Chromosomal DNA from *P. gingivalis* 33277 was used as the template for cloning purposes. The construction of *P. gingivalis* 33277-derived strains expressing various GFP fusion proteins is described in Text S1.

### Site-directed mutagenesis and construction of a mutated HBP35 protein expression system

Site-directed mutagenesis was performed using a QuickChange Lighting Site-Directed Mutagenesis kit (Stratagene, La Jolla, CA, USA). To create mutated HBP35 protein with M^1^A, M^135^A, Y^291^A, G^295^A, K^296^A, G^324^A, Y^326^A, or K^340^A, the oligonucleotide primer pairs, M1AFw/M1ABw, M135AFw/M135ABw, Y291AFw/Y291ABw, G295AFw/G295ABw, K296AFw/K296ABw, G324AFw/G324ABw, Y326AFw/Y326ABw, or K340AFw/K340ABw were used, respectively, with the recombinant plasmid pKD817 as the template. To create mutated HBP35 with K^340^A, CPG70 with K^817^A and PAD with K^553^A, the oligonucleotide primer pairs K340AFw/K340ABw, K817AFw/K817ABw and K553AFw/K553ABw were used, respectively, with the recombinant plasmid pKD770, pKD796 or pKD797 as the template. Each KpnI-NotI fragment containing an appropriate mutation was digested and inserted into the same region of pTCB [40]. Each pTCB vector containing the amino acid substitution was introduced into *E. coli* S17-1 by electroporation, and then the transformant was conjugated with *P. gingivalis* 33277 or KDP166 [*hbp35* deletion mutant] [10] and selected on TS agar plates containing Gm and Tc. To create mutated HBP35 protein with K^273^A, the oligonucleotide primer pairs, K273AFw/K273ABw was used with the recombinant plasmid pKD755 [10] as the template for site-directed mutagenesis, giving rise to pKD820. pKD820 DNA linearized with SacI was introduced into KDP166 (Δ*hbp35*) by electroporation [10], yielding a strain with replacement of Δ*hbp35* by *hbp35*(K^273^A).

### Gel electrophoresis and immunoblot analysis

*P. gingivalis* cells were lysed in SDS sample buffer, and cell lysates were separated by SDS-PAGE and transferred onto polyvinylidene fluoride (PVDF) membranes. Blots were blocked with 5% BSA in TBS (PBS containing 0.5% Tween 20) in the case of anti-A-LPS or with 3% skim milk in TBS in the case of anti-HBP35, anti-GFP or anti-CTDs for 1 h at room temperature and probed with antibodies at appropriate dilutions at 4°C overnight. Antigen-antibody binding was detected with anti-rabbit or mouse IgG peroxidase conjugate (1∶ 2000, Dako, Japan) and the ECL substrate (GE Healthcare).

### Antibodies

The mAb 1B5 [11] was used as anti-A-LPS. The preparation of anti-HBP35 [41] and anti-TprA [26] has been described previously. mAb Pg-ompA2 [42] was used as an anti-HBP35 monoclonal antibody. An anti-GFP monoclonal antibody (JL-8) was purchased from Clontech. Purified recombinant prolyl tripeptidyl aminopeptidase A (PtpA) consisting of L^39^-D^730^ [43] was kindly given by Dr. T. Yoshimoto (Nagasaki University, Japan). To raise an anti-PtpA antibody, rabbits were immunized with the PtpA protein and antiserum against PtpA was collected from the immunized rabbits at Biomedical Research Center, Center for Frontier Life Sciences in Nagasaki University. Animal care and experimental procedures were performed in accordance with the Guidelines for Animal Experimentation of Nagasaki University with approval of the Institutional Animal Care and Use Committee (approval number 0707020606). To obtain mouse antisera against peptides, three peptides corresponding to the amino acid regions of CTD1 ([A^274^-V^288^] within HBP35), CTD2 ([P^286^-E^300^] within HBP35) and CTD3 ([E^301^-L^315^] within HBP35), each with a cysteine residue synthesized at its N-terminus, were constructed and conjugated to keyhole limpet hemocyanin (Sigma Genosys). Mouse antisera against the synthetic peptides were purchased from EveBioscience Co., Ltd. (Wakayama, Japan).

### Dot blot analysis

Dot blot analysis was performed as described previously [44], with some modifications. Briefly, *P. gingivalis* cells that had fully grown in enriched BHI medium were harvested, washed with PBS and suspended with PBS. The washed cells were adjusted to an OD_595_ of 0.5. Three microliters of the adjusted cells was blotted directly onto a nitrocellulose membrane and left to dry.

### Preparation of periplasmic fractions

Periplasmic fractions were prepared from *P. gingivalis* by a method described previously [45]. After being suspended in 50 mM Tris acetate buffer (pH 7.8) containing 0.75 M sucrose, *P. gingivalis* cells were treated with lysozyme (final concentration 0.1 mg/ml) on ice for 2 min. Conversion to spheroplasts was performed by slowly diluting the cell suspension over a period of 10 min with 2 volumes of cold 1.5 mM EDTA. After centrifugation at 10,000× g for 10 min, the resulting supernatant was used as the periplasmic fraction.

### Immuno-electron microscopy with anti-HBP35-conjugated gold particles

Processed nickel grids were loaded with the processed *P. gingivalis* strains for 1 min. The grids were subsequently blocked with a blocking solution for goat gold conjugate (Aurion) for 5 min at room temperature and then incubated in diluted anti-HBP35 for 30 min. After washing the primary antibody, the grids were incubated for 30 min at room temperature in immuno-gold goat anti-rabbit IgG (Aurion) diluted 1∶30 in incubation buffer. Staining was done with a 0.5% uranyl acetate solution after washing the secondary antibody, and stained samples were visualized with a JEM-1230 transmission electron microscope (JEOL, Japan).

### Immunoprecipitation

*P. gingivalis* cells were harvested and the cell pellets were dissolved with BugBuster (Novagen) in the presence of TLCK and leupeptin. The samples were then immunoprecipitated with protein G agarose beads (Amersham) with anti-rabbit HBP35 polyclonal antibody.

### LC-MS/MS and cell fractionation analysis

Sample preparation and all procedures were performed as described previously [10], [25].

## Supporting Information

Table S1 Identification of peptides from the diffuse HBP35 protein and the 40-kDa HBP35 protein by LCMS.
(XLS)

Table S2 Strains used in this study [37]–[39].
(XLS)

Table S3 Plasmids used in this study [40].
(XLS)

Table S4 Primers used in this study.
(XLS)

Text S1 Construction of *P. gingivalis* strains expressing various GFP fusion proteins and *P. gingivalis* mutant strains.
(DOC)
