# Correction: Por Secretion System-Dependent Secretion and Glycosylation of *Porphyromonas gingivalis* Hemin-Binding Protein 35

**DOI:** 10.1371/journal.pone.0203154

**Published:** 2018-08-29

**Authors:** Mikio Shoji, Keiko Sato, Hideharu Yukitake, Yoshio Kondo, Yuka Narita, Tomoko Kadowaki, Mariko Naito, Koji Nakayama

There is an error in the fourth sentence under the “Anti-HBP35 immunoblot analysis of various mutants nonreactive to anti-A-LPS” heading of the Results section. The correct sentence is: We found in this study that PGN_0242 (encoding a putative mannosyl transferase), PGN_1056 (*vimA*), PGN_1236 (*porR*), PGN_1242 (*wzy*), PGN_1251 (*gtfB*), PGN_1255 (*rfa*) and PGN_1302 (*waaL*) mutants all show nonreactivity to an anti-A-LPS antibody (Fig 4A).

There is an error in the mutant strain name in Fig 4. Please see the correct Fig 4 here.

**Fig 4:**
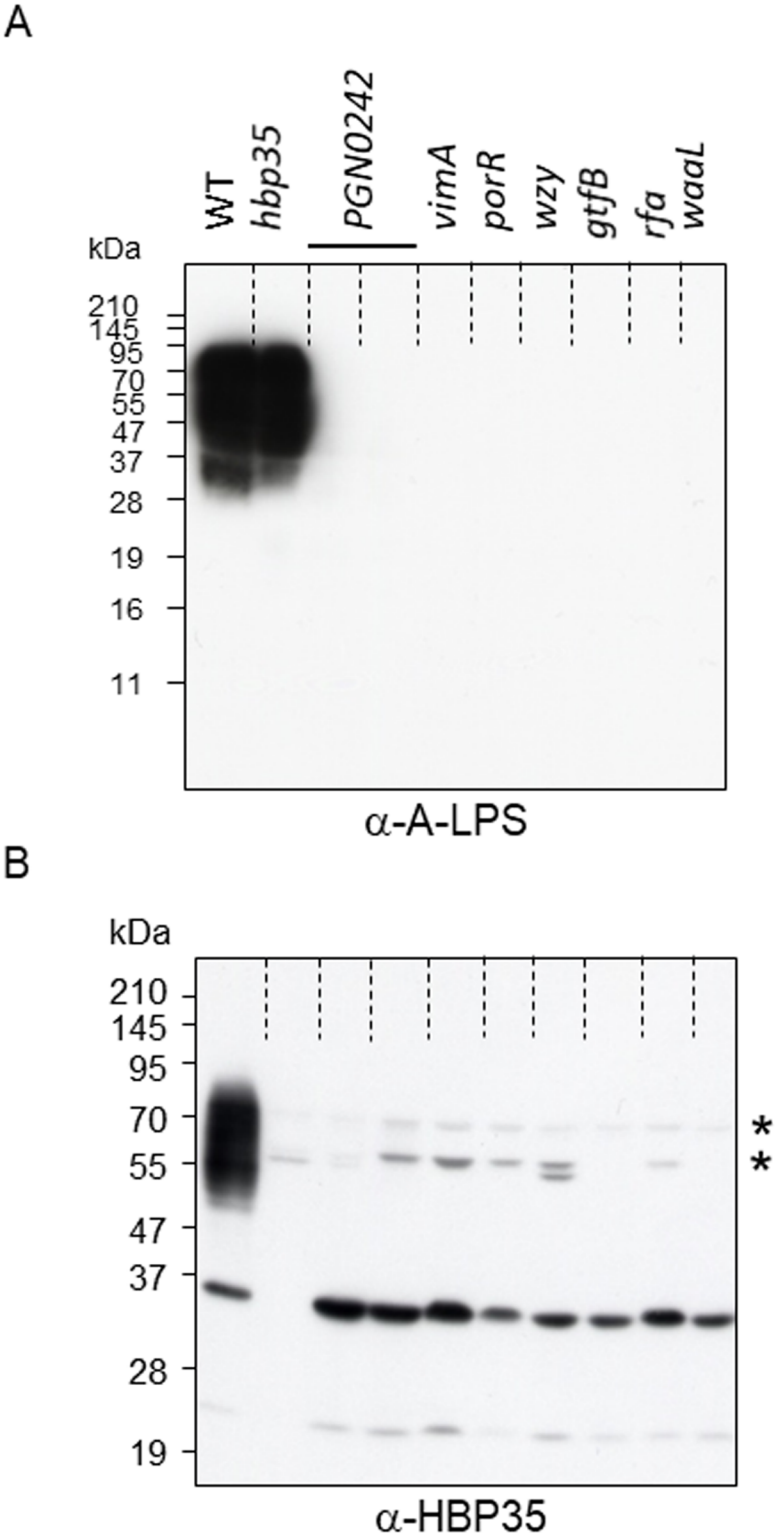
Immunoblot analysis of *P*. *gingivalis* A-LPS biosynthesis-deficient mutants with anti-A-LPS and anti-HBP35 antibodies. Cell lysates of *P*. *gingivalis* A-LPS biosynthesis-deficient mutants were subjected to SDS-PAGE and immunoblot analysis with anti-A-LPS and anti-HBP35 antibodies. Asterisks indicate nonspecific cross-reactive protein bands.
