# Supplementary material for: Por Secretion System-Dependent Secretion and Glycosylation of Porphyromonas gingivalis Hemin-Binding Protein 35
Source: PLoS One. 2011 Jun 22;6(6):e21372. doi: 10.1371/journal.pone.0021372 (PMC3120885; doi:10.1371/journal.pone.0021372)
Supplement: Text S1 — Construction of P. gingivalis strains expressing various GFP fusion proteins and P. gingivalis mutant strains. (DOC) [file pone.0021372.s005.doc]

**Text S1**

**Construction of *P. gingivalis* strains expressing various GFP fusion proteins**

The amplified fragment was digested with appropriate restriction enzymes and inserted into the same sites of a vector. To construct the GFP fusion vectors which contain a GFP with a signal sequence region of HBP35 in its N-terminus and a C-terminal region of HBP35 in its C-terminus, a 0.30-kb PCR fragment encoding the promoter region of *hbp35* gene and N-terminal region (M^1^-G^32^) of HBP35 from *P. gingivalis* 33277 was amplified using a primer pair, HBPsignalFw/HBPsignalBw, and then cloned into pCR4.0 to yield pKD768. A 0.71-kb PCR fragment encoding a GFP from pGFPuv (Clonteck) was amplified using a primer pair, GFPuvFw/GFPuvBw, and then cloned into pCR4.0 to yield pKD769. C-terminal HBP35 region DNA with the terminator was amplified from the *P. gingivalis* 33277 chromosomal DNA using primer pairs HBP260Fw/HBPTerBw for CTD[HBP35(N^260^-P^344^)], HBP265Fw/HBPTerBw for CTD[HBP35(N^265^-P^344^)], HBP286Fw/HBPTerBw for CTD[HBP35(P^286^-P^344^)], HBP303Fw/HBPTerBw for CTD[HBP35(Y^303^-P^344^)], HBP313Fw/HBPTerBw for CTD[HBP35(G^313^-P^344^)], HBP318Fw/HBPTerBw for CTD[HBP35(N^318^-P^344^)], HBP323Fw/HBPTerBw for CTD[HBP35(P^323^-P^344^)], HBP327Fw/HBPTerBw for CTD[HBP35(V^327^-P^344^)], HBP329Fw/HBPTerBw for CTD[HBP35(R^329^-P^344^)], HBP334Fw/HBPTerBw for CTD[HBP35(G^334^-P^344^)], HBP340Fw/HBPTerBw for CTD[HBP35(K^340^-P^344^)] and HBPStopFw/HBPTerBw for Stop, and then cloned into pCR4.0, to yield pKD770 to pKD781, respectively. The KpnI-EcoRI region of pKD768 containing the 0.30-kb PCR fragment was inserted into the same region of pBluescript SK-, resulting in pKD782. The EcoRI-PstI region of pKD769 was inserted into the same region of pKD782 to yield pKD783. The PstI-NotI regions of pKD770 to pKD781 were inserted into the same region of pKD783, to yield pMS17 to pMS28, respectively. The KpnI-NotI regions of pKD784 to pKD795 were inserted into the same region of pTCB [40], to yield pKD835 to pKD846, respectively. The plasmids pKD835 to pKD846 were introduced into *E. coli* S17-1 by transformation [38], and then the transformants were conjugated with the *P. gingivalis* wild-type and *porT* strains and selected on the TS agar plates containing Gm and Tc to yield WT/GFP-CTD[HBP35(N^260^-P^344^)], WT/GFP-CTD[HBP35(N^265^-P^344^)], WT/GFP-CTD[HBP35(P^286^-P^344^)], WT/GFP-CTD[HBP35(Y^303^-P^344^)], WT/GFP-CTD[HBP35(G^313^-P^344^)], WT/GFP-CTD[HBP35(N^318^-P^344^)], WT/GFP-CTD[HBP35(P^323^-P^344^)], WT/GFP-CTD[HBP35(V^327^-P^344^)], WT/GFP-CTD[HBP35(R^329^-P^344^)], WT/GFP-CTD[HBP35(G^334^-P^344^)], WT/GFP-CTD[HBP35(K^340^-P^344^)] and WT/GFP, and *porT*/GFP-CTD[HBP35(N^260^-P^344^)], *porT*/GFP-CTD[HBP35(N^265^-P^344^)], *porT*/GFP-CTD[HBP35(P^286^-P^344^)], *porT*/GFP-CTD[HBP35(Y^303^-P^344^)], *porT*/GFP-CTD[HBP35(G^313^-P^344^)], *porT*/GFP-CTD[HBP35(N^318^-P^344^)], *porT*/GFP-CTD[HBP35(P^323^-P^344^)], *porT*/GFP-CTD[HBP35(V^327^-P^344^)], *porT*/GFP-CTD[HBP35(R^329^-P^344^)] and *porT*/GFP-CTD[HBP35(G^334^-P^344^)] strains, respectively. To construct GFP fusions with CTD[CPG70], CTD[PAD], CTD[P27] and CTD[RgpB], CTD[CPG70]-, CTD[PAD]-, CTD[P27]- and CTD[RgpB]-encoding regions were cloned into pCR4 using primer pairs, CPGFw/CPGBw, PADfw/PADBw, P27Fw/P27Bw and RgpBFw/RgpBBw to yield pKD796 to pKD799, respectively. The PstI-NotI regions of pKD796 to pKD799 were inserted into the same region of pKD783 to yield pKD809 to pKD812, respectively. The KpnI-NotI regions of pKD809 to pKD812 were inserted into the same region of pKD859 to pKD862, respectively. Plasmids pKD859 to pKD862 were intoduced into *E. coli* S17-1, and then the transformants were conjugated with the *P. gingivalis* wild-type and *porT* strains, and then selected on the TS agar plates containing Gm and Tc to yield WT/GFP-CTD[CPG70], WT/GFP-CTD[PAD], WT/GFP-CTD[P27], *porT*/GFP- CTD[CPG70], *porT*/GFP-CTD[PAD] and *porT*/GFP-CTD[P27].

**Construction of *P. gingivalis* mutants**

A 2.3-kb DNA fragment containing the *vimA* and *recA* genes was cloned into pUC19 by suppression of the hypersensitivity of an *E. coli recA* mutant (UM255) [39] against cumen hydroperoxide, to yield pKD833. Since there was one BglII site within the *vimA* gene in pKD833, the *tetQ* gene cassette derived from pKD375 [37] was inserted into the BglII site of pKD833, to yield pKD834. pKD834 DNA linearized with BspEI was introduced into *P. gingivalis* 33277 by electroporation to yield a *vimA* mutant, KDP202. To create *P. gingivalis* mutants *porV* (KDP361), *PGN_0663* (KDP201), *wzy* (KDP204) and *waaL* (KDP203), regions upstream of the genes were amplified with primer pairs PGN0023upFw/PGN0023upBw, PGN0063upFw/PGN0063upBw, PGN1242upFw/PGN1242upBw and PGN1302upFw/PGN1302upBw, respectively and were cloned into the pGEM-T easy vector. Regions downstream of the genes were amplified with primer pairs PGN0023dwFw/PGN0023dwBw, PGN0063dwFw/PGN0063dwBw, PGN1242dwFw/PGN1242dwBw and PGN1302dwFw/PGN1302dwBw, respectively and were cloned into the pGEM-T easy vector. The each upstream and downstream regions were inserted into the appropriate restriction sites within pBluescript II SK(-). And then, a 1.5-kb BamHI *ermF* gene cassette was inserted into the BamHI or BglII site of those recombinant plasmids. The resulting targeting vectors pKD866, pKD867, pKD868 and pKD869 were digested with BssHII or NotI and were introduced into *P. gingivalis* 33277 by electroporation.
